# Association of adverse pregnancy outcomes and intimate partner violence survivorship: a cross-sectional survey

**DOI:** 10.3389/fgwh.2026.1616403

**Published:** 2026-02-10

**Authors:** Wah Wah Myint, Samia Tasnim, Saylor Mealing, Aniyah Zaman, Chimuanya Osuji, Gogoal Falia, Matthew Lee Smith

**Affiliations:** 1 Center for Community Health and Aging, School of Public Health, Texas A&M University, College Station, TX, United States; 2 Department of Health Behavior, School of Public Health, Texas A&M University, College Station, TX, United States; 3 Department of Epidemiology and Biostatistics, School of Public Health, Texas A&M University, College Station, TX, United States; 4 Department of Health Policy and Management, School of Public Health, Texas A&M University, College Station, TX, United States; 5 Center for Health Equity and Evaluation Research, School of Public Health, Texas A&M University, College Station, TX, United States

**Keywords:** adverse pregnancy outcomes, intimate partner violence, Jordan, social determinants, women

## Abstract

**Introduction:**

Adverse pregnancy outcomes (APOs) are poorly understood among women who experienced intimate partner violence (IPV). This study examines the influence of lifetime IPV experiences and social determinants on APOs among Jordanian married women.

**Methods:**

Cross-sectional data was examined from 4,419 women in the 2023–2024 Jordan Family and Population Health Survey. The outcome variables were APOs, LBW, and pregnancy loss (e.g., miscarriages, stillbirths). The exposure variable was lifetime IPV. Covariates were social determinants (age, education, wealth quintile, residency, regions), having children aged ≤ 5, delivering a singleton or twins/multiple births, using a skilled birth attendant (SBA), and being smokers. Descriptive and bivariate analyses were performed, and a series of binary logistic regression was fitted, controlling for the covariates.

**Results:**

About 9.5% (*n* = 377) of women reported miscarriages, 0.8% (*n* = 45) induced abortions, 0.2% (*n* = 14) reported stillbirths, and 89.5% reported live births. Among the live births (*n* = 3,983), 23.7% had a baby born with LBW. Of the sample, 6.7% (*n* = 289) reported experiencing IPV. Of them, 86.6% (*n* = 245) reported delivering a live birth baby, 13.25% (*n* = 43) reported miscarriage/abortion, 0.1% (*n* = 1) reported stillbirths. Logistic regression results showed that women from the richest wealth quintile group [adjusted odds ratio (aOR) = 0.50] and those who used an SBA (aOR = 0.07) had lower odds of reporting APOs compared to their counterparts. Contrarily, the individuals living in the Northern region showed higher odds of APOs (aOR = 1.43) compared to those that live in the Central region. Among IPV victims, those in the rural areas had higher odds of APOs and LBW infants (aOR = 7.72, *p* = 0.001), those from the Southern region had lower odds of APO (aOR = 0.14, *p* = 0.030), those than the reference categories. All *p*-values are <0.05.

**Discussion:**

Findings highlight the need for additional research related to the pregnancy implication of women with a history of IPV, especially among those living in the Jordan's Northern region and those from poorer wealth quintile.

## Introduction

Adverse pregnancy outcomes (APOs) affect millions of women worldwide. APOs can include pregnancy loss or complications (1). Pregnancy loss includes stillbirth (i.e., babies born with no sign of life at 28 weeks of pregnancy or later), miscarriage (i.e., spontaneous or missed abortion), and induced abortion (i.e., ending pregnancies by medical or surgical means) (1). Pregnancy complications could be babies born with low birth weight (LBW) (i.e., weighed lower than 2.5 kilograms at the time of birth), born preterm also known as preterm birth (PTB) (i.e., born before 37 completed weeks), and born smaller or larger than average for their gestational age (1). The prevalence of APOs varies depending on the type of APOs and geography.

In 2020, about 10% of babies had PTB. In 2021, stillbirths accounted for approximately 31% of global cases, while in 2023, about 61% of women had unintended pregnancies, and 29% of all pregnancies ended in induced abortions (2, 3). In the Middle Eastern countries, there were 20.83 stillbirths per 1,000 births, 15.7% LBW, and 10.9% PTB in 2020 (3). Compared to regional estimates, Jordan showed a higher percentage of LBW at 18.9% but lower stillbirth rates at 9.13 per 1,000 births in 2020 (3).

Women experiencing APOs have increased risk of mortality and long-standing health ramifications (4). There are many direct and indirect causes of APOs including abortion, maternal and child morbidity, and mortality (5). Some of the known risk factors for APOs are social determinants including maternal age, education, socio-economic status, ethnicity, employment, multiple pregnancy, inadequate use of antenatal care services, and smoking (4–6). For example, both younger (i.e., <20 years) and aged ≥35 years) maternal ages have been shown to increase the risk of APOs, specifically stillbirth, fetal growth restriction, and neonatal death (7). Women who were unemployed had higher chances of PTB and LBW compared to the women in employment (8).

Because every pregnancy carries potential risk and the need for changes to prepare familial unit, both future parents can experience mental strain that can impact their relationship (9). In some unfortunate situations, this could contribute to physical and/or emotional altercations between partners. Despite what is known about the risk factors associated with APOs, less is known about the potential role that intimate partner violence (IPV) plays in pregnancy outcomes (10).

### Intimate partner violence (IPV)

Intimate partner violence (IPV) is one of the most pervasive and under-reported public health problems around the world. According to the World Health Organization, IPV refers to “behavior by an intimate partner or ex-partner that causes physical”, sexual or psychological harm, including physical aggression, sexual coercion, psychological abuse, and controlling behaviors (11). The IPVs could also be different forms within each type; For example, within physical IPV, there could be less severe or more severe form (12). The controlling behavior can be in the form of economic (13) or controlling free movement (i.e., limiting women to see, visit, and interact with others) (14). IPV can also include stalking, i.e., repeated unwanted attention and contact by an intimate partner igniting fear and safety concerns (15).

Globally, one-in-three women experienced some form of violence within their lifetimes (11). Perpetrators of IPV come from diverse socioeconomic, cultural, and religious backgrounds, leaving all women at potential risk (12). In many countries, legal systems and cultural norms often normalize IPV, leading to inadequate protection and support for victims. This is particularly evident in the Middle East and North African (MENA) countries, where studies indicate that between 6% and 59% of women have experienced physical IPV, 3% to 40% have experienced sexual IPV, and 5% to 91% have suffered from emotional IPV (13). Specifically, a previous umbrella review study found that the prevalence of any type of IPV during pregnancy was as high as 44.1% in the MENA countries (16).

Intimate partner violence (IPV) has far-reaching consequences for women's overall health and social well-being (17). The impacts of IPV include serious physical injuries and increased risk of chronic health conditions such as anxiety, depression, post-traumatic stress disorder (PTSD), gastrointestinal problems, cardiovascular diseases, sexually transmitted infections including Human Immunodeficiency Virus (HIV), and unintended pregnancies (18–20). Women experiencing IPV often engage in high-risk behaviors such as smoking and substance abuse, while simultaneously facing social isolation, fear of retaliation, coercion by abusers, financial dependence, and significant barriers to accessing essential healthcare services (21). Additionally, many women who experienced IPV victimization received poor social support (22), despite it was known that social support can lessen the severity of IPV at least (23).

Societal factors contributing to the high prevalence of IPV include social norms that condone violence against women, gender inequality in both political and economic spheres, and legal impunity for perpetrators. Individual risk factors include women's acceptance of violence as a husband's right, young age, and infrequent communication with their original family (24–28). Also, prior literature identified IPV and APO risk factors such as education (29), poverty (30), place of residence, region, use of skill birth attendants, number of ANC visit, having children under age 5 years, parity, smoking (31–33), and presence of sexually transmitted infections (STI) (34, 35). The physical, psychological, emotional, sexual, economic, and social manifestations of IPV significantly affect maternal health and frequently result in APOs (36–39).

In Jordan, IPV remains a substantial issue despite notable progress in gender parity, women's empowerment, and women's health (40). A nationally representative survey in Jordan revealed that the lifetime prevalence of IPV was 25.9%, with emotional violence being the most common (20.6%), followed by physical violence (17.5%) and sexual violence (5.1%) (40). It was also reported that fatal IPV cases contributed to 33% of all the femicide cases in Jordan (41). A prior study from Jordan reported that women living in urban areas of Amman have a higher incidence of IPV, with potential reasons of stress associated with urban lives among the perpetrators (42). A recent World Health Organization (WHO) report also indicated that 24% of ever-partnered women in Jordan have experienced some forms of IPV during their lifetime (11). Additionally, there were studies that identified risk factors for IPV among Jordian women including intergenerational violence (43) or having number of children, but examination on APOs outcome was lacking (44). Also, another study in Jordan examined the disparities negative birth outcomes, however, IPV exposure was lacking (45). However, very rare study focuses on APOs based on IPV exposure.

Although most research concentrates on the adverse effects of IPV during pregnancy, there is substantial limitation of research on a lifetime IPV victimization and APOs in Jordan context. For example, Abujiban and colleagues identified an association between physical IPV and higher risks of APOs (i.e., pregnancy-induced hypertension, cesarean section, more pain killer use during birth, and excessive use of postnatal medication) using a hospital-based sample (46). However, their study did not find any significant association between physical IPV and other factors regarding pregnancy plan/care (unplanned pregnancy, inadequate ANC visits (i.e., less than eight), or birth outcomes (abortions, premature rupture of membranes, PTB, induction of labor, episiotomy, laceration, or postpartum hemorrhage) (46). Also, Afkhamzadeh and colleagues examined APOs based on IPV during pregnancy, identification on any lifetime IPV (i.e., not necessarily only the time of pregnancy) was lacking (47).

All these inconclusive and inconsistent findings on different APOs suggest a need to investigate the relationship between lifetime IPV and various types of APOs (21). Understanding the connection between a lifetime IPV exposure and APOs can inform prevention and intervention strategies aimed at reducing these risks. Despite the high prevalence of IPV in Jordan, the link between the lifetime IPV and different types of APOs (i.e., pregnancy loss, pregnancy complications including LBW, PTB) are poorly understood, which could vary depending on a country's culture and context or region. Our hypothesis was that those who experienced lifetime IPV are at increased risk of any type of APOs. As such, the purpose of this study was to examine the influence of lifetime IPV experiences and social determinants on APOs among Jordanian married women.

### Linkages between APOs and IPV

As per Harman's trauma theory when people experienced traumatic events such as IPV, they have subsequent negative health issues (48, 49). These health issues include PTSD (50, 51), cognitive problems (4), insomnia (51–53), worsening night vision during pregnancy (54), higher level of somatic problems, and confusion (55), which all can contribute to APOs. Trauma history has substantial effects on prenatal anxiety and stress (56), which could subsequently lead to microsomic babies (51, 57) or pregnancy loss (58). For example, prior scholars had identified that the major stress around conception had impact on infants including conotruncal heart defects or isolated cleft palate, or neural tube defects (59). Selye's theory of stress postulated that stress can be caused by diverse stimuli regardless of the events (60). And the response to the stress differed based on individuals and stages of stress (i.e., alarm reaction, resistant stage, and exhaustion stage) (60).

Women can face IPV at any time despite the frequency, types, and extent are varied (61). For example, a meta-analysis identified that the IPV prevalence was highest before pregnancy, lower during pregnancy, and higher again after childbirth (61). Additionally, Walker's cycle of violence theory posited that women can be revictimized repeatedly as women's motivation to respond reduced over time, however, these repeated victimizations subsequently contribute stress and anxiety throughout their life (62).

A study by Garabedian and colleagues identified that history of any IPV victimization (i.e., sexual, physical, or stalking) had significant association with increased risk of PTB, pre-eclampsia, or bleeding during third trimester (63). Specifically, their study found that sexual IPV victimization with or without other IPVs was associated with all these APOs, those with physical IPV victimization had bleeding and PTB, and those with stalking experience had pre-eclampsia (63). Additionally, Bonomi and colleagues identified that those with history of IPV victimization had higher risk of depression, anxiety, substance use, or other chronic diseases, reproductive system-related conditions such as menstrual disorders and sexually transmitted infections, all of which can negatively impact on their future pregnancies (64). IPV victimization can also be a barrier for perinatal health services used (17, 42). For example, a study in Ethiopia found that almost 23.6% of pregnant women were restricted by their partners for their ANC use during their pregnancy (65). Additionally, a recent study in Egypt identified that almost 58.8% of women reported psychological IPV victimization were found to be associated with delayed ANC, experienced hemorrhage, and premature rupture of membrane (66). Another scoping review identified an association between delayed ANC and any type of IPV (despite the study did not specify timing of IPV victimization event) (67).

Along with inadequate ANC, IPV victimization during pregnancy is associated with miscarriage or complications such as insufficient weight gain, premature rupture of membrane, placental abruption, placenta previa, PTB, hemorrhage, uterine rupture, and maternal death. IPV victimization during the perinatal period may increase the risk of PTB, LBW infants, and stillbirths (18). A systematic review and meta-analysis of seventeen studies found that IPV victimization during pregnancy were three times more likely to suffer perinatal death in comparison to those who did not (17). Considering the seriousness of the adverse impacts of IPV victimization, researchers have further explored the association between various types of IPV victimization and APOs. Physical and sexual IPV victimization was significantly associated with PTB, LBW, and increased hospitalization, while emotional IPV victimization was associated with PTB (18–20).

More broadly, IPV victimization has been linked to pregnancy health, various maternal complications, and reduced utilization of adequate ANC, all of which contribute to different types of APOs (68, 69), and therefore, understanding risk factors specifically among IPV victims are important and this study is needed.

## Materials and methods

### Data source

**Figure 1:**
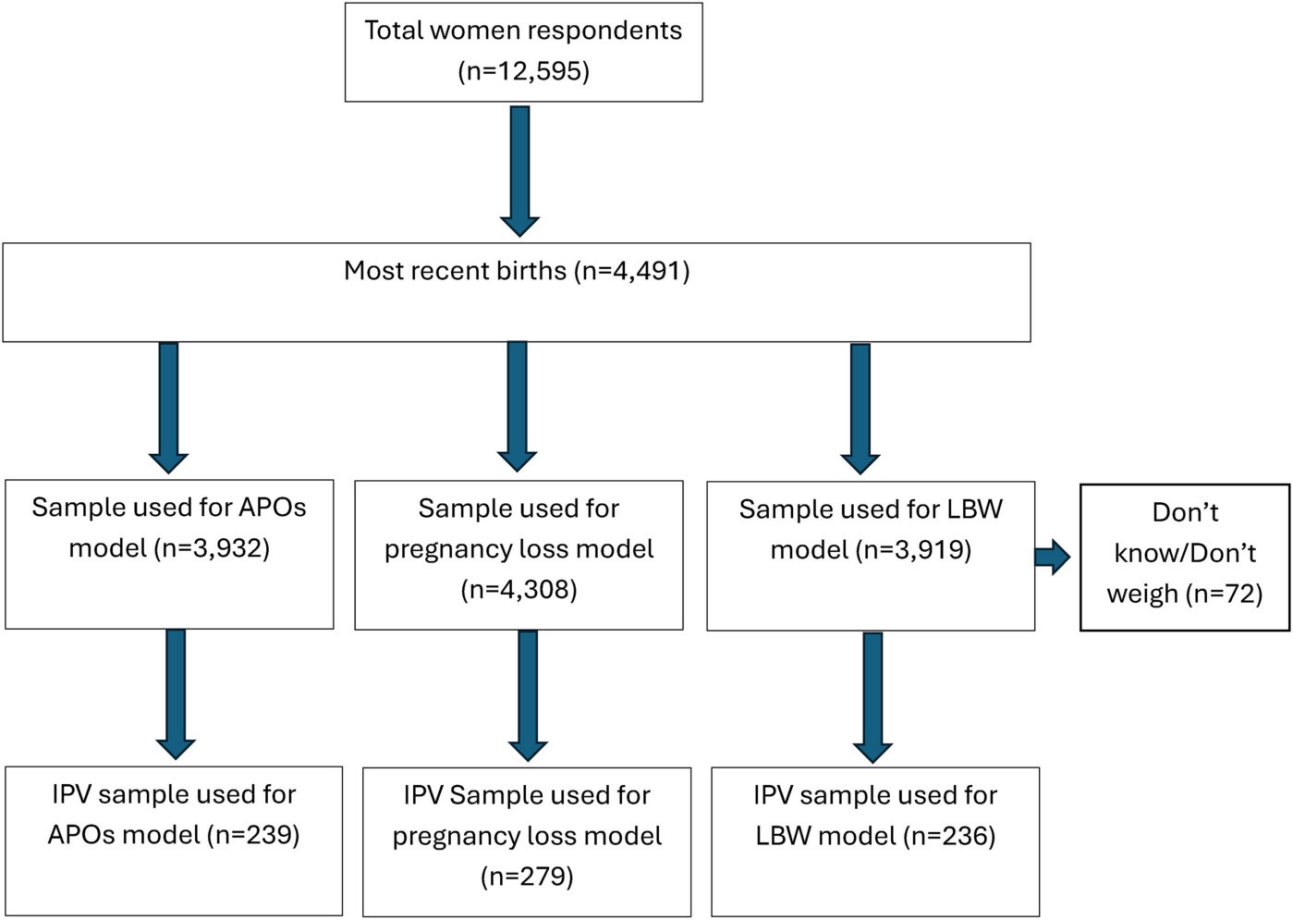
Sample flow chart.

This study used deidentified respondents’ data from women in the Jordanian Population and Family Health Survey (JPFHS), which was implemented from January to June 2023 (37). The JPFHS utilized a two-stage stratified random sampling design: First, households were randomly selected based on the 2016 Jordan's census framework, then, a participant from each selected household was randomly chosen and interviewed (37). The JPFHS included ever-married women aged 15–49 years (*n* = 12,595) with a response rate of 97% (37). For this study, the women who did not report their most recent pregnancy outcomes (i.e., recent live births, stillbirths, miscarriages, and induced abortions), those who reported don't know, and/or those who mentioned that their babies were not weighted (*n* = 69), and those who reported not knowing if their babies were too small or big (*n* = 3) were excluded from analyses, resulting in a final sample of 4,419 women (Figure 1).

### Variables

#### Outcome variable

The main outcome variable was APOs, a composite variable created from four original variables, which were then dichotomized. The first variable was based on the item “pregnancy outcome declared by respondent”. Response choices were born alive, born dead, miscarriage, and abortion. We assigned “no = 0” for the response “born alive” and “yes = 1” for the other responses of stillbirths, miscarriages, or induced abortions. The second variable identified if the respondents had babies born with LBW. We categorized “yes = LBW” (scored 1) if the baby's birth weight was <2.5 kg and “no = otherwise” (scored 0). The third variable used was “size at birth”, with response choices being “very large, larger than average, average, smaller than average, and very small”. We scored cases who responded “average” as 0, meaning there were no APOs. We scored cases who responded that their babies were macrosomia (i.e., very large, larger than average) and microsomia (i.e., smaller than average, or very small) as 1 because this indicates APOs. The fourth variable used was PTB, which was defined as “the baby born prior to 9 months”. The JPFHS program collected birth data in months (i.e., calendar months) for the PTB (22). Despite the exact PTB definition referring to a baby born alive prior to 37 weeks of gestation, we refer to “baby born prior to 9 months when we refer to PTB throughout the manuscript” (22–24). Cases reporting PTB were scored 1 and those without PTB as 0. Then, we summed the four APOs variables together to create a composite score. We assigned “yes” (scored 1) for those who reported “yes” to any of the four APOs variables, and we assigned “no” (scored 0) for those who reported “no” for all four variables.

#### Exposure variable

The main exposure variable was the experience of any type of IPV victimization in the respondents' lifetime (22). It was created from four main variables: emotional IPV victimization (no, yes), any less severe physical IPV victimization (no, yes), any severe physical IPV victimization (no, yes), and sexual IPV victimization (no, yes).

The emotional IPV victimization included has ever been humiliated, threatened with harm, insulted, or made to feel bad, ignored or neglected, sexually ignored or rejected, threatened, or kicked out of the house by the partner. The less severe physical IPV victimization includes being pushed, shook, or had something thrown, slapped, punched with fist or hit by something hard, had an arm twisted, or hair pulled by the partner. Severe physical IPV victimization included being kicked or dragged, strangled, or burnt, attacked with a knife/gun or other weapons by the partner. Sexual IPV included ever been physically forced into unwanted sex, ever been forced into other unwanted sexual acts, and physically forced to perform sexual acts respondent didn't want to. Then, we summed the four variables together to create a composite score. We assigned “yes” (scored 1) for those who reported “yes” to any of the four IPV victimization variables, and we assigned “no” (scored 0) for those who reported “no” for all four IPV victimization variables.

#### Covariates

##### Sociodemographic variables

The sociodemographic variables included the women's age in a five-year group (i.e., 15–19, 20–24, 25–29, 30–34, 35–39, 40–44, 45–49), education level (i.e., no education, primary, secondary, and higher), current employment (i.e., yes, no), wealth index in quintiles (i.e., poorest, poorer, middle, richer, richest), which are based on distribution of the household population. The wealth quintile is a composite variable which is calculated based on household characteristics (i.e., materials used for housing constructions, source of drinking water, toilet facilities), assets (i.e., ownership of televisions, bicycles, or car), types of access to water, and sanitation facilities (70, 71). We also included places of residence (i.e., urban, rural) and regions (i.e., North, Central, and South). The reference groups for these variables were “age group 15–19 years”, “having no education”, “being in the poorest wealth quintile”, “living in rural areas”, and “those living in the Northern region”, respectively.

##### Other important risk factors

We also included other important risk and protective factors such as number of children under 5 (i.e., no children, 1–2 children, ≥3 children), child born was twin or more (i.e., no = not twin/multiple, yes = twin/multiple), number of ANC visits (<8 visits, and ≥8 visits), use of an SBA, such as doctors, nurses, and midwives (i.e., no, yes), presence of STI, whether the individuals smoked either cigarettes or tobaccos (i.e., no, yes), whether the individuals thought that getting medical help for themselves or the distance to health facility was a big problem (i.e., no, yes), and the involvement of others in decision making for health care use (i.e., respondent alone, respondent and her partner, her partner alone, or other people). The reference groups were “having no children under 5 years old” and “no” for other variables, respectively.

### Statistical analysis

Stata standard edition (SE) 19.0 was used for all the analyses (72). For descriptive statistics, univariate and bivariate [i.e., Pearson's Chi-square (*χ*^2^) test of independence] analyses were performed with the analytic sample (*n* = 4,419). The Pearson's *χ*^2^ test is used to investigate the relationship between outcome variables and covariates (73). Only *p-*values of ≤0.05 were considered statistically significant.

Prior to analysis, we assessed multicollinearity among covariates by calculating variance inflation factors (VIF) (Supplementary Tables S1 and S2). Multicollinearity was assessed separately for the full sample and the subset of those who experienced IPV. Mean VIF (for the full sample) was within the acceptable range, indicating no serious multicollinearity (i.e., VIF greater than 5–10 is considered multicollinearity present) (74, 75). In the subset of IPV victims, higher VIF was observed. Nonetheless, based on the mean VIF, we included the covariates in our subset analyses (74, 75).

A series of binary logistic regression models were performed: (a) to predict the odds ratio of overall APOs, LBW, and pregnancy loss; and (b) to examine the relationship between the dependent variables and covariates. All socio-demographic variables and exposure variables were included guided by prior literature (4, 29–35), regardless of their statistically significant levels in bivariate analyses. Subgroup analyses were performed with samples limited to those who experienced IPV victimization (APOs, LBW, and pregnancy) [Note. For PTB, Micro/macro-somia, and pregnancy loss, subset analyses were not performed due to their limited sample sizes]. We used complete case analyses for our prediction models, i.e., missingness was not included. The detailed sample flow is shown in the sample flow-chart (Figure 1).

Additionally, we calculated marginal effects to quantify the effect of change on the outcome variables (Tables 2, 3) including overall APOs (Model 1), LBW (Model 1a) and pregnancy loss (Model 1b) among all sampled women. Moreover, we performed subgroup marginal effects analysis for APOs-Model 2, LBW (Model 2a, and pregnancy loss-Model 2b) among women who experienced IPV victimization.

All weighted analyses (i.e., descriptive, bivariate, logistic regression, and marginal analyses) were performed applying survey weights to account for complex sampling design (i.e., clustering, stratification, and sampling weights), as recommended by the Demographic and Health Survey Program (37–39). If we do not apply survey weights that account for complex sampling design, the calculation of point estimates and their standard errors will be incorrect. Also, lack of consideration on clustering can lead to underestimation of standard errors (76). Therefore, we applied “svy” commands using Stata software, which accounts for sampling weights, clustering, and stratification (77). For the sensitivity analysis, we evaluated differences in prediction estimates by comparing weighted and unweighted binary logistic regression models (Supplementary Tables S1 and S2) (78).

## Results

Of the 4,419 women in the sample, about 89.5% (*n* = 3,983) reported giving live births, 9.5% (*n* = 377) reported miscarriages, 0.8% reported induced abortion (*n* = 45), and 0.2% (*n* = 14) reported stillbirths. Among those who reported live births, 23.7% (*n* = 1,015) reported LBW, while 26.5% (*n* = 1,164) reported macrosomia or microsomia. Among the 4,491 participants, 289 (6.7%) reported experiencing any IPV victimization. Of them, 12.2% (*n* = 40) reported miscarriages, 1.0% (*n* = 3) reported induced abortions, 0.1% (*n* = 1) reported stillbirths, and 86.6% (*n* = 245) reported delivering live births. Among those who experienced IPV victimization and delivered live births, 24.4% (*n* = 67) reported LBW, 32.7% (*n* = 87) reported macrosomia or microsomia, and 4.6% (*n* = 11) reported preterm birth. Among women who gave birth to a LBW infant, 6.6% (*n* = 67) reported experiencing any IPV victimization. Of those who reported PTB, 9.5% (*n* = 10) reported a history of IPV victimization, and of those who reported macrosomia or microsomia, 8.0% (*n* = 87) reported a history of any IPV victimization.

Table 1 shows bivariate results showing the proportion of APOs among different groups. A larger proportion of women who were aged 30–34 (*χ*^2^ = 66.55, *p* < 0.001), had secondary education levels (*χ*^2^ = 46.34, *p* = 0. 001), were in the poorest groups (*χ*^2^ = 47.51, *p* = 0.005), and lived in the Northern region (*χ*^2^ = 11.90, *p* < 0.038), reported experiencing APOs and had a child or two (*χ*^2^ = 95.32, *p* < 0.001) reported experiencing APOs. Interestingly, a larger proportion of APOs were seen among those who gave birth to a single child (*χ*^2^ = 21.40*, p* = 0.002). Interestingly, we did not find any significant association between APOs and IPV (*χ*^2^ = 1.69, *p* = 0.411). [Note: *χ*^2^ represents uncorrected *χ*^2^, and *p*-values are from Rao-Scott adjusted *χ*^2^ test].

**Table 1:** Results from bivariate analysis (column percentage).

Variables	No: No APOs (*n* = 2,248)	Yes: APOs reported (*n* = 2,171)	Total (*n* = 4,419)	*χ* ^2^	*P*-value
	*n* (weighted %)	*n* (weighted %)	*n* (weighted %)		
Age in 5 years groups				66.55	0.003
15–19	49 (2.1)	61 (2.9)	110 (2.5)		
20–24	326 (13.0)	322 (15.0)	648 (13.9)		
25–29	636 (30.2)	533 (23.4)	1,169 (27.1)		
30–34	613 (27.0)	544 (25.3)	1,157 (26.3)		
35–39	443 (19.8)	449 (20.4)	892 (20.0)		
40–44	167 (7.2)	214 (10.3)	381 (8.7)		
45–49	14 (0.7)	48 (2.7)	62 (1.6)		
Education				46.34	0.001
No education	59 (1.8)	45 (1.6)	104 (1.7)		
Primary	163 (5.0)	203 (8.0)	366 (6.3)		
Secondary	1,227 (53.5)	1,282 (59.6)	2,509 (56.3)		
Higher	799 (39.7)	641 (31.0)	1,440 (35.7)		
Wealth quintile				47.51	0.005
Poorest	747 (23.7)	854 (29.8)	1,601 (26.5)		
Poorer	527 (22.5)	493 (24.4)	1,020 (23.4)		
Middle	489 (23.1)	419 (20.7)	908 (22.0)		
Richer	347 (17.8)	298 (17.3)	645 (17.6)		
Richest	138 (13.0)	107 (7.9)	245 (10.7)		
Current employment				0.03	0.913
No	2,036 (90.4)	1,965 (90.3)	4,001 (0.4)		
Yes	212 (9.6)	206 (9.7)	418 (9.6)		
Place of residence				7.77	0.062
Urban	1,854 (90.6)	1,746 (88.0)	3,610 (89.4)		
Rural	394 (9.4)	425 (12.0)	824 (10.6)		
Region				11.90	0.038
Central	918 (64.0)	755 (59.2)	1,673 (61.8)		
North	851 (29.8)	970 (34.6)	1,821 (32.0)		
South	479 (6.2)	446 (6.2)	925 (6.2)		
Number of children under 5				95.31	<0.001
No child	105 (6.4)	252 (15.1)	360 (10.5)		
1–2 children	1,761 (79.1)	1,600 (73.9)	3,368 (76.6)		
≥ 3 children	382 (14.5)	319 (11.0)	706 (12.1)		
Child born is twin or multiple				21.39	0.002
No, single birth	2,229 (99.1)	2,048 (97.3)	4,277 (98.3)		
Yes	19 (0.9)	70 (2.7)	89 (1.7)		
Number of ANC visit				6.80	0.069
<8 visits	876 (35.3)	740 (39.4)	1,616 (36.9)		
≥8 visits	1,368 (64.7)	1,004 (60.6)	2,372 (63.1)		
Use of skilled birth attendants¶				4.95	0.161
No	1 (0.1)	4 (0.2)	5 (0.1)		
Yes, Doctors	1,506 (65.6)	1,162 (66.7)	2,668 (67.8)		
Yes, nurses	741 (31.4)	583 (33.1)	1,324 (32.1)		
STI				0.55	0.619
No	2,022 (92.3)	1,968 (92.9)			
Yes	196 (7.79)	181 (7.2)	377 (7.5)		
Smoking cigarettes or tobacco				11.93	0.054
No	2,057 (89.7)	1,972 (86.3)	4,029 (88.2)		
Yes	191 (10.3)	199 (13.7)	390 (11.8)		
Big problem for medical help or distance to health facility				2.12	0.318
No	1,850 (86.5)	1,789 (85.0)			
Yes	398 (13.5)	382 (15.0)			
Decision making				7.78	0.351
Respondent alone	480 (23.9)	467 (23.7)	947 (23.87)		
Respondent and her partner	1,505 (69.8)	1,519 (67.8)	3,124 (68.9)		
Her partner alone	154 (6.1)	179 (8.2)	333 (7.1)		
Other people	4 (0.2)	3 (0.3)	790.2)		
Any IPV					
No	2,119 (93.8)	2,011 (92.8)	4,130 (93.3)	1.69	0.411
Yes	129 (6.2)	160 (7.2)	289 (6.7)		

IPV refers to intimate partner violence.

¶=The use of skilled birth attendants-related questions was answered only by 3,997 respondents.

Counts were unweighted frequencies. The percentages were adjusted accounting for the complex survey design.

χ^2^ denotes Chi-square. The χ^2^ are uncorrected Pearson's χ^2^.

*P*-values were from Rao-Scott adjusted χ^2^ tests accounting for the complex survey design.

### Factors associated with any APO—full sample

Supplementary Table S1 presents the weighted results of the binary logistic regression model identifying factors associated with APOs, LBW, and pregnancy loss. The women from the middle quintile to upper wealth quintile groups were less likely to experience APOs, compared to those from the poorest wealth quintile group, with the lowest odds ratio was observed among the richest wealth quintile group [adjusted odds ratio (aOR) = 0.50, *p* = 0.007]. Those living in the Northern region showed a higher odds ratio (aOR = 1.44, *p* = 0.001) of experiencing APOs compared to those living in the Central region. Additionally, women who gave birth to twins or multiples were more likely to experience APOs compared to those who delivered a singleton baby (aOR = 3.68, *p* = 0.001). Those who used SBAs were less likely to experience APOs compared to those who did not (aOR = 0.06, *p* = 0.020). Our model regarding among all sampled women showed that there is no significant association between the IPV victimization and APOs.

### Factors associated with LBW– full sample

As reported in the Supplementary Table S1, among those who give livebirths (*n* = 3,919), higher odds of LBW babies were seen among women residents of the Northern region (aOR = 1.38, *p* = 0.009) and those who give birth to twins or multiple children (aOR = 5.48, *p* < 0.001). However, lower odds ratios of LBW babies were seen among those who were from the richer (aOR = 0.58, *p* = 0.011), and richest (aOR = 0.48, *p* = 0.23) wealth quintile groups, and those who used an SBA (aOR = 0.03, *p* = 0.002) compared to their reference categories.

### Factors associated with pregnancy loss– full sample

As reported in the Supplementary Table S1, among the covariates, being in older age groups had higher odds of pregnancy loss, and the highest odds ratio was seen among those from aged 44–45 group (aOR = 66.47, *p* < 0.001), those who had a smoking history had higher odds of pregnancy loss compared to those who did not smoke (aOR = 1.63, *p* = 0.039). Contrarily, women with two or more children who were under 5 years old had lower odds of pregnancy loss compared to those who did not have children under 5 years old (aOR = 0.05, *p* < 0.001).

For the sensitivity analyses among all sampled women, we compared weighted and unweighted aORs. The detailed results of unweighted aORs of APOs, LBW, and pregnancy loss among all sampled women are described in Supplementary Table S1. The results of unweighted aORs were somewhat similar to the weighted aORs for APOs except for two variables, i.e., region and use of SBA. Regarding the region, we found higher odds ratio of APOs among women Southern residents (unweighted aOR = 1.32, *p* = 0.003). Additionally, we found no significant relationship between APOs and use of an SBA in unweighted model. Regarding LBW, the results were somewhat similar, and the weighted aORs were lower than unweighted aORs except the variable “region” (unweighted aOR = 1.33, *p* = 0.001). In the unweighted model with the pregnancy loss outcome, our study finding identified that the aORs were quite lower compared to the weighted models. For example, despite the highest odds were seen among women from the 45–49 age group (unweighted aOR = 16.01, *p* < 0.001), the odds ratio was somewhat lower than that of the weighted model.

Marginal analyses of APOs, LBWs, and pregnancy loss of all sampled women are described in Table 2. Our study identified that compared to the women from the reference categories, lower likelihood of APOs was seen among women from the middle wealth quintile group (*β* = −0.08, *p* = 0.017), those from the richest wealth quintile (*β* = −0.16, *p* = 0.004), and those who used an SBA (*β* = −0.52, *p* < 0.001). Contrarily, higher likelihood of APOs was seen among women from the Northern region (*β* = 0.09, *p* = 0.001) and those who have twins or more birth (*β* = 0.31, *p* < 0.001) compared to women from the reference categories. Among all sampled women, compared to the women from the reference categories, lower likelihood of LBW was observed among women from the richer (*β* = −0.09, *p* = 0.009) and those from the richest wealth quintile group (*β* = −0.12, *p* = 0.008), and those who used an SBA (*β* = −0.69, *p* < 0.001). Contrarily, higher likelihood of LBW was observed among those who had twins or more (*β* = 0.39, *p* < 0.001) and residents of the Northern region (*β* = 0.06, *p* = 0.010), compared to those from the reference categories. The women from all older age groups were more likely to have pregnancy loss compared to those from 15 to 19 age groups, and the greatest probability of pregnancy loss was seen among those from 45 to 49 age groups (i.e., 28 percentage point more likely to have pregnancy loss).

**Table 2:** Results from marginal analysis of adverse pregnancy outcomes (APOs), low birth weight (LBW), and pregnancy loss (all sampled women).

Variables	Model 1: APOs (*n* = 3,932)		Model 1a: LBW (*n* = 3,919)		Model 1b: Pregnancy loss (*n* = 4,308)	
	*β* (95% CI)	SE	β (95% CI)	SE	β (95% CI)	SE
Age in 5 years groups						
15–19	Ref.					
20–24	−0.04 (−0.22, 0.15)	−0.10	−0.09 (−0.24, 0.06)	−0.08	0.04 (0.01, 0.07) **	−0.01
25–29	−0.11 (−0.29, 0.07)	−0.09	−0.11 (−0.25, 0.04)	−0.07	0.03 (0.01, 0.05) **	−0.01
30–34	−0.06 (−0.24, 0.13)	−0.10	−0.06 (−0.2, 0.09)	−0.07	0.06 (0.04, 0.09) ***	−0.01
35–39	−0.08 (−0.27, 0.11)	−0.10	−0.05 (−0.2, 0.11)	−0.08	0.09 (0.06, 0.12) ***	−0.01
40–44	−0.02 (−0.22, 0.18)	−0.10	−0.05 (−0.2, 0.10)	−0.08	0.13 (0.08, 0.18) ***	−0.03
45–49	0.05 (−0.27, 0.37)	−0.16	−0.05 (−0.29, 0.19)	−0.12	0.28 (0.11, 0.45) **	−0.09
Education						
No education	Ref.					
Primary	0.11 (−0.09, 0.32)	−0.10	0.09 (−0.07, 0.26)	−0.08	0.01 (−0.08, 0.09)	−0.04
Secondary	0.03 (−0.15, 0.21)	−0.09	0.02 (−0.13, 0.17)	−0.08	0.00 (−0.08, 0.08)	−0.04
Higher	−0.01 (−0.20, 0.17)	−0.09	−0.02 (−0.17, 0.14)	−0.08	−0.03 (−0.11, 0.05)	−0.04
Wealth quintile						
Poorest	Ref.					
Poorer	−0.03 (−0.10, 0.04)	−0.04	−0.01 (−0.07, 0.05)	−0.03	−0.01 (−0.03, 0.02)	−0.01
Middle	−0.08 (−0.15, −0.01)*	−0.04	−0.05 (−0.12, 0.01)	−0.03	0.00 (−0.03, 0.03)	−0.01
Richer	−0.08 (−0.17, 0.00)	−0.04	−0.09 (−0.16, −0.02)**	−0.04	0.02 (−0.02, 0.05)	−0.02
Richest	−0.16 (−0.27, −0.05)**	−0.06	−0.12 (−0.21, −0.03)**	−0.05	−0.02 (−0.05, 0.02)	−0.02
Place of residence						
Urban	Ref.					
Rural	0.04 (−0.03, 0.12)	−0.04	0.02 (−0.04, 0.08)	−0.03	0.01 (−0.03, 0.04)	−0.02
Region						
Central	Ref.					
North	0.09 (0.03, 0.14)**	−0.03	0.06 (0.01, 0.10)**	−0.02	−0.01 (−0.03, 0.01)	−0.01
South	0.04 (−0.02, 0.11)	−0.03	0.02 (−0.03, 0.06)	−0.02	−0.01 (−0.03, 0.01)	−0.01
Currently employed						
No	Ref.					
Yes	0.06 (−0.04, 0.16)	−0.05	0.04 (−0.05, 0.12)	−0.04	0.03 (−0.02, 0.07)	−0.02
Number of children under age 5 years						
No child	Ref.					
One or two children	−0.02 (−0.13, 0.09)	−0.06	−0.01 (−0.1, 0.09)	−0.05	−0.3 (−0.38, −0.22)***	−0.04
Three or more children	−0.08 (−0.21, 0.04)	−0.06	−0.01 (−0.12, 0.09)	−0.06	−0.33 (−0.41, −0.25)***	−0.04
Child born is twins or multiple						
No, single birth	Ref.					
Yes	0.31 (0.15, 0.48)***	−0.08	0.39 (0.21, 0.57)***	−0.09	0.07 (−0.04, 0.18)	−0.06
Number of ANC visit						
<8 visits	Ref.					
≥8 visits	−0.04 (−0.08, 0.01)	−0.02	−0.03 (−0.07, 0.01)	−0.02	NA	NA
Use of skilled birth attendants						
No						
Yes	−0.52 (−0.71, −0.33)***	−0.1	−0.69 (−0.87, −0.51)***	−0.09	NA	NA
Presence of STI in the past 12 months	Ref.					
No						
Yes	−0.02 (−0.11, 0.06)	−0.04	−0.02 (−0.09, 0.05)	−0.03	0.02 (−0.02, 0.07)	−0.02
Smoking cigarettes or tobacco						
No	Ref.					
Yes	0.07 (−0.02, 0.16)	−0.05	0.00 (−0.08, 0.07)	−0.04	0.03 (0.00, 0.07)	−0.02
Big problem for medical help or distance to health facility						
No	Ref.					
Yes	−0.02 (−0.09, 0.05)	−0.04	0.01 (−0.06, 0.07)	−0.03	0.02 (−0.02, 0.05)	−0.02
Decision making						
Respondent alone	Ref.					
Respondent and her partner	0.01 (−0.05, 0.08)	−0.03	0.00 (−0.05, 0.05)	−0.03	−0.01 (−0.04, 0.01)	−0.01
Her partner alone	0.07 (−0.04, 0.18)	−0.05	0.03 (−0.06, 0.13)	−0.05	0.00 (−0.04, 0.05)	−0.02
Other people	0.14 (−0.40, 0.68)	−0.27	0.18 (−0.39, 0.74)	−0.29	−0.04 (−0.11, 0.03)	−0.04
Any IPV						
No	Ref.					
Yes	0.01 (−0.09, 0.11)	−0.05	0.00 (−0.08, 0.08)	−0.04	0.00 (−0.03, 0.03)	−0.02

APOs, adverse pregnancy outcomes; LBW, low birth weight; β, coefficient; SE, standard error; CI, confidence interval.

* *p* < 0.05.

** *p* < 0.01.

*** *p* < 0.001.

### Factors associated with any APO—subset of IPV victims

As presented in Supplementary Table S2, among IPV victims, those in the rural area had higher odds of APOs (aOR = 7.72, *p* = 0.001) compared to those living in the urban area, however, those in the Southern regions had lower odds of APOs (aOR = 0.14, *p* = 0.030) compared to those living in the Central region.

### Factors associated with LBW—subset of IPV victims

As presented in Supplementary Table S2, among IPV victims, higher odds of LBW were seen among those had primary education (aOR = 50.7, *p* = 0.010), secondary education (aOR = 23.92, *p* = 0.047), rural residents (aOR = 15.04, *p* < 0.001), those who had three or more children under age 5 years (aOR = 13.66, *p* = 0.055), and those whose health decisions were made by others (aOR = 7.21, *p* = 0.008) compared to those in the reference categories. Contrarily, lower odds of LBW were seen among those who were from middle (aOR = 0.20, *p* = 0.021) and richer wealth quintile groups (aOR = 0.15, *p* = 0.018), and those who had ANC ≥8 visits (aOR = 0.21, *p* = 0.001).

### Factors associated with pregnancy loss– subset of IPV victims

As presented in Supplementary Table S2, among IPV victims, the higher odds of pregnancy loss were seen those who were from older age groups compared to those 15–19 age groups, among the women residents of Southern region (aOR = 6.09, *p* = 0.039), who had twins or multiple births (aOR = 66.15, *p* = 0.001), those who were from the poorer wealth quintile, and those who had children under age 5 years compared to the reference categories.

For the sensitivity analyses among the IPV victims, we compared weighted and unweighted effect sizes (i.e., aORs). The detailed results of unweighted aORs of APOs, LBW, and pregnancy loss among women IPV victims are described in Supplementary Table S2. In the model with APOs outcome, those living in the rural areas had higher odds (aOR = 7.72, *p* < 0.001) in the weighted model, compared to unweighted model (aOR = 2.57, *p* = 0.026). Despite the significant relationship between APOs and Southern region being identified in the weighted model (aORs = 0.14, *p* = 0.026), we saw significant relationship between APOs and Northern region in the unweighted model (aOR = 2.11, *p* = 0.024). Additionally, although we did not see any significant association between APOs and decision making in the weighted model, a significant association between decision making by partner only and APOs (aOR = 2.66, *p* = 0.048) was found in the unweighted model.

In the weighted model with LBW, despite seeing significant association between LBW and primary or secondary education, we did not find any significant relationship between LBW and education in the unweighted model. Also, there were differences in significant association between LBW and wealth quintiles between weighted and unweighted model. Additionally, although we found significant association between LBW and place of residence in both models, the odds ratio was higher in weighted model (aOR = 15.04, *p* < 0.001) compared to that of unweighted model (aOR = 3.69, *p* = 0.003). The results of pregnancy loss were slightly different. All age groups, wealth quintile, region, the number of children under age 5 years old, showed lower odds in the weighted model compared to the unweighted model. Despite we found significant association between twins or multiple births with pregnancy loss (aOR = 66.15, *p* = 0.010) in the weighted model, we did not see any significant association in the unweighted model.

The results from marginal analyses among IPV victims are presented in Table 3. Our findings identified that higher likelihood of APOs was observed among rural residents compared to the urban residents (*β* = 0.47, *p* < 0.001). Contrarily, women from the Southern region had lower likelihood of APOs (*β* = −0.46, *p* = 0.034) compared to the residents from the Central region. The higher likelihood of delivering LBW babies was found among women who had primary education (*β* = 0.43, *p* = 0.011) or who had secondary education (*β* = 0.35, *p* = 0.032) compared to those who had no formal education. Similarly, higher likelihood of delivering LBW infants was observed among women who had 3 or more children under age 5 years (*β* = 0.29, *p* = 0.018), those living in the rural (*β* = 0.30, *p* = 0.001), and those with other people who made the pregnant women health care decision (*β* = 0.22, *p* = 0.009) compared to the reference categories. Contrarily, women from middle (*β* = −0.18, *p* = 0.028) and richer (*β* = −0.21, *p* = 0.029) wealth quintiles, and those who had ANC≥8 visits (*β* = −0.17, *p* < 0.001) were less likely to have LBW babies. Additionally, our findings also showed that higher likelihood of pregnancy loss was seen among those who had twins or more compared to women with singleton delivery (*β* = 0.62, *p* = 0.013). Contrarily, lower likelihood of pregnancy loss was observed among the poorer (*β* = −0.09, *p* = 0.014) or richer (*β* = −0.08, *p* = 0.041) wealth quintile groups, and those who had three or more children under age 5 years (*β* = −0.38, *p* = 0.037) compared to the respective reference categories.

**Table 3:** Results from marginal analysis among women IPV survivors.

Variables	Model 2: APOs (*n* = 239)		Model 2a: LBW (*n* = 236)		Model 2b: pregnancy loss (*n* = 279)	
	β (95% CI)	SE	β (95% CI)	SE	β (95% CI)	SE
Age in 5 years groups						
15–19						
20–24	−0.25 (−1.17, 0.66)	−0.46	−0.20 (−0.94,0.55)	−0.38		–
25–29	−0.20 (−1.11, 0.71)	−0.46	−0.11 (−0.83,0.61)	−0.36		–
30–34	−0.13 (−1.08, 0.81)	−0.48	−0.12 (−0.85,0.62)	−0.37		–
35–39	−0.03 (−0.93, 0.87)	−0.45	−0.02 (−0.75,0.71)	−0.37		–
40–44	−0.61 (−1.53, 0.31)	−0.47	−0.30 (−1.08,0.47)	−0.39		–
45–49	−0.10 (−1.26, 1.07)	−0.59	–			–
Education						
No education						
Primary	0.06 (−0.39, 0.51)	−0.23	0.43 (0.10, 0.76)*	−0.17	0.01 (−0.05, 0.06)	−0.03
Secondary	0.06 (−0.34, 0.47)	−0.21	0.35 (0.03, 0.66)*	−0.16	0.01 (−0.04, 0.06)	−0.02
Higher	−0.23 (−0.68, 0.23)	−0.23	0.18 (−0.16,0.52)	−0.17	0.03 (−0.04, 0.11)	−0.04
Wealth quintile						
Poorest	−0.02 (−0.26, 0.22)	−0.12	0.04 (−0.07,0.15)	−0.06	−0.09 (−0.16, −0.02)*	−0.04
Poorer	−0.32 (−0.65, 0.00)	−0.16	−0.18 (−0.33, −0.02)*	−0.08	0.02 (−0.09, 0.14)	−0.06
Middle	−0.26 (−0.72, 0.20)	−0.23	−0.21 (−0.39, −0.02)*	−0.09	−0.08 (−0.15, 0.00)*	−0.04
Richer	−0.18 (−0.7, 0.35)	−0.27	0.07 (−0.17,0.31)	−0.12	0.02 (−0.17, 0.21)	−0.09
Richest						
Place of residence						
Urban						
Rural	0.47 (0.22, 0.73)***	−0.12	0.3 (0.12, 0.47)**	−0.09	0 (−0.04, 0.04)	−0.02
Region						
Central						
North	0.16 (−0.05, 0.37)	−0.11	0.07 (−0.06,0.2)	−0.07	0.04 (−0.01, 0.09)	−0.02
South	−0.46 (−0.88, −0.03)	−0.21	−0.2 (−0.48,0.09)	−0.15	0.09 (−0.06, 0.23)	−0.07
Currently employed						
No						
Yes	0.28 (−0.08, 0.63)	−0.18	0.21 (−0.02,0.44)	−0.11	−0.02 (−0.05, 0.01)	−0.02
Number of children under age 5 years						
No child						
One or two children	0.20 (−0.21, 0.6)	−0.20	0.16 (−0.07,0.39)	−0.12	−0.36 (−0.71, 0)*	−0.18
Three or more children	0.29 (−0.15, 0.74)	−0.23	0.29 (0.05–0.52)*	−0.12	−0.38 (−0.73, −0.02)*	−0.18
Child born is twins or multiple						
No, single birth						
Yes	–		–		0.62 (0.13, 1.10)*	−0.25
Number of ANC visit						
<8 visits						
≥8 visits	−0.20 (−0.41, 0)	−0.1	−0.17 (−0.26, −0.08)***	−0.05	NA	–
Use of skilled birth attendants						
No						
Yes	–		–		NA	–
Presence of STI in the past 12 months						
No						
Yes	0.14 (−0.16, 0.43)	−0.15	0.12 (−0.02–0.25)	−0.07	−0.03 (−0.05, 0)	−0.01
Smoking cigarettes or tobacco						
No						
Yes	−0.24 (−0.55, 0.08)	−0.16	−0.09 (−0.3–0.11)	−0.10	0.04 (−0.03, 0.1)	−0.03
Big problem for medical help or distance to health facility						
No						
Yes	−0.06 (−0.28, 0.15)	−0.11	−0.06 (−0.21–0.09)	−0.08	0.02 (−0.04, 0.08)	−0.03
Decision making						
Respondent alone						
Respondent and her partner	0.12 (−0.1, 0.35)	−0.11	0.1 (−0.01–0.21)	−0.05	0.02 (−0.01, 0.05)	−0.02
Her partner alone	0.23 (−0.07, 0.52)	−0.15	0.22 (0.06–0.38)**	−0.08	0.02 (−0.04, 0.08)	−0.03
Other people	NA		NA		NA	

APOs, adverse pregnancy outcomes; LBW, low birth weight; β, coefficient; SE, standard error; CI, confidence interval.

* *p* < 0.05.

** *p* < 0.01.

*** *p* < 0.001.

## Discussion

The present study examined the social determinants of APOs and the influence of IPV experiences on APOs among Jordanian married women. The study found that of the women who experienced APOs, 6.7% also experienced IPV victimization. In regression models, APOs were associated with a variety of socio-demographic variables, including wealth, region of residency, number of living children, using an SBA, and pregnancy of multiples, specifically twins. Higher wealth, having one to two living children, and using an SBA at the time of childbirth were all protective against APOs.

The finding that being part of higher wealth quintile groups (i.e., middle, richer, and richest) was protective from experiencing APOs is in alignment with previous studies on APOs. Previous studies have found that lower maternal socioeconomic status or poverty level is associated with an increased risk of experiencing APOs, such as PTBs, stillbirths, and abortions (79, 80). A lack of income or wealth historically indicates a lack of access to antenatal care, as lower income levels are often associated with a lack of health insurance (81–84).

Furthermore, limited opportunity for health literacy, limited awareness of the benefits of using an SBA, and a lack of means to attend healthcare appointments decrease the chances of a mother receiving adequate antenatal care, thus increasing the chances of APOs (79, 85–88). Additionally, mothers who had one to two living children had a lower incidence of APOs, which may be associated with the stress of first-time pregnancy or caring for more children while pregnant. Antenatal stress is associated with an increased risk of APOs, and previous studies have found that mothers tend to be more stressed during their first pregnancies and when they are caring for multiple children while pregnant (87, 89). The use of an SBA during delivery was associated with a decreased risk of APOs, which is aligned with several previous studies, as the birthing attendant is better able to monitor the symptoms and signs of APOs compared to someone without their skills and knowledge base (35, 36).

These socio-demographic variables were all protective factors against APOs, with being in the wealthiest quintile and using an SBA being the most protective. Additionally, in the current study, residency in the Northern region of Jordan and twin births were identified as risk factors for APOs. Moreover, living in the Northern region of Jordan was associated with a higher incidence of APOs in our study. The Northern region of Jordan is home to many populations, including a large population of Syrian refugees, who experience a high number of barriers to healthcare access, utilization, and implementation, as well as sexual and gender-based violence and have higher rates of APOs, in comparison to Jordanian women (45, 68, 69). The combination of the lack of access to healthcare contributes to APOs, in that there is delayed or no care, which increases the chances of a mother or infant having APOs (90). The presence of multiples, specifically twin or multiple pregnancy in this study, was found to increase the incidence of APOs, which has also been seen in previous studies (84, 91, 92). Higher prevalences of APOs, such as pre-eclampsia, PTB, and infant mortality, have been found in twin births when compared to singleton births (84, 93). The increased incidence of APOs is due to the stress of multiple fetuses on the mother's uterus, insufficiency of the placenta, and insufficiency of the cervix (81, 82). These risk factors heavily influenced the APOs.

In the binary logistic regression of the overall sample, we observed null significant finding between APOs and IPV, which could be many different reasons. One of the reasons could be temporal mismatch of IPV, i.e., we did not specify the timing of IPV victimization in our analysis. Despite the information on experience of violence victimization during pregnancy was collected by JDHS, only 3% of ever-married women disclosed about their violence victimization by anyone including their husband (37) leading the sample size too small. Therefore, we combined all different types of IPV. Combining all types of IPV may also contribute to the null findings. For example, a prior study found an association between physical IPV and APOs, but emotional IPV (i.e., fear) was not associated with APOs (94). Secondly, the overall IPV sample is small, which may have been an issue to establish significant association (95). Additionally, despite a recent WHO report indicated that 24% of every-partner women in Jordan have experienced some forms of IPV victimization during their lifetime (5), our study revealed only 6.7%. This may have been under-reporting due to social desirability bias and may be the cause for null findings. Finally, it is also possible that there could be APOs associated with prior pregnancy, which may strain in the relationship and results in any or all types of IPV. All these ambiguities suggest a need for further investigation.

In all models of sampled women (especially APOs and LBW), our findings revealed that the odds ratio of the unweighted models are higher than the weighted models. Perhaps the unweighted models may be overestimated and may have been biased (96, 97). As we applied complex survey about weight accounting for design and sampling, our models' odds ratio may reflect an accurate result, minimizing these biases (96, 97). However, we identified a different pattern for the model with the outcome pregnancy loss. We observed that the weighted aORs of many variables that showed significant relationship were higher than unweighted aORs. Therefore, further investigation into this pattern is needed. Perhaps, caution might be taken when interpreting pregnancy loss.

The findings from marginal analyses among overall sampled women were somewhat aligned with the findings from the logistic regression results. Unsurprisingly, those in the richer or richest wealth quintile group or those who used an SBA had lower likelihood of APOs or LBW. Perhaps, those in the wealthier wealth quintiles may have access to health care access or those who used an SBA have access to health information (98). Also, the fact that higher likelihood of LBW was observed among those who had twins or multiple births had higher likelihood of LBW (84, 93) and residents of the Northern region may have been linked to specific population. The women from all older age groups were more likely to have pregnancy loss compared to those from 15 to 19 age groups, and the greatest probability of pregnancy loss was seen among those from 45 to 49 age groups, which also is a known factor.

### Among women with IPV victimization experiences

Overall, the prevalence of lifetime IPV victimization was 6.7%. IPV victimization increases during pregnancy due to the stress and instability that can be brought on by pregnancy and the prospect of raising a child (83). Worldwide prevalence of IPV during pregnancy ranges between 2% and 67%, with one study finding that when several forms of IPV combined were reported, the prevalence of any kind of IPV was 25%, and another finding that some countries have rates as high as 67% (48–50, 99–101). Experiencing IPV across the life span, including the pregnancy period, negatively impacts the mother's health across her life span (102–107) as well as the birth outcomes (11, 108). Therefore, it is critical to detect IPV early and prevent further health consequences.

Among those IPV victims, women in the rural area had higher odds of APOs compared to those living in the urban areas. This finding is partially aligned with a prior study in which the urban residents had lower odds of pregnancy loss (109). The societal acceptance of IPV in the rural areas may create undisclosed or repeated violence, that could lead to APOs. For example, a prior study in Jordan had revealed that men living in the villages had higher acceptance of attitude toward emotional and physical violence (110) which might influence their perpetration as normal, leading to higher perceived societal acceptance on violence, creating stressful situation for women (83), which could further lead to higher APOs. Or women's lack of decision making may create delayed health care or limited ANC visits (111). It is also possible that health care workers missed screening the IPV during the ANC. For example, a study in the US (despite geographic differences) showed that there was a higher chance of not screening for IPV in the rural areas during the ANC visits (111). All the prior research and our research findings suggest an urgent need to ensure early and routine ANC, and IPV screening for every health care visit including before, during, and after pregnancy for every woman (111), specifically to the women in the rural area. Interestingly, our findings revealed that those in the Southern region had lower odds of APOs compared to those in the Central region of Jordan. Although not exactly the same, the prior study in Jordan identified that the Central region had a higher risk for LBW, one of the pregnancy complications (112). Perhaps the women in the Central region may have less social support, especially among those who had IPV, putting them stressful situation which could lead them to experience APOs. All these mixed findings suggest a need for further investigation.

Among the IPV victims, higher odds of LBW were seen among those who had primary or secondary education compared to those who did not have any education. Despite a prior study in the US that found that mothers' illiteracy as the risk factor for LBW (113), our study found that those who have primary or secondary education had higher risk of LBW compared to those who did not have formal education. Perhaps these contradictory findings may be due to geographic differences or the way variables were defined, requiring further investigation. Additionally, a prior study in Jordan found that education is negatively associated with LBW babies. Perhaps, education facilitates better health service use including use of ANC or an SBA (98, 114). All these inconsistent findings suggest further investigation. Also, among the IPV victims living in rural areas had higher odds of LBW support the fact that the societal perception of acceptance of violence (83) or lack of social support (115) may create undisclosed stressful conditions for the women, leading them experiencing pregnancy complications.

Those who had three or more children under age 5 years compared to those who did not have children under 5 had a higher odd of LBW. This may also have been related to inadequate use of ANC due to potential restrictions of health services used by partners (65) or lack of social support (115). Not surprisingly, the individuals whose health care service use was decided by others compared to those who made their own decisions had higher odds of APOs. For example, as stated elsewhere, ANC use could be delayed due to the restriction from their spouses (65) or if they were traumatized due to their IPV victimization experiences (116). Adequate ANC used are important as health care providers can identify potential risks, IPV screening, and make appropriate action to minimize the risk. As expected, and widely known, our study identified that those who were from the higher wealth quintile groups (especially from the middle and richer wealth quintile groups) had lower odds of delivering LBW babies. Our findings regarding the association between the wealth quintile and LBW among IPV victims contradicts with a prior study conducted in South Africa, in which IPV victims from the rich wealth quintiles were more likely to have LBW babies, suggesting further investigation between wealth and LBW association, considering other stressful and traumatic factors. Additionally, our study confirmed that those who had ANC ≥8 visits have lower odds of LBW infant delivery.

Our findings regarding the higher odds of pregnancy loss among IPV victims, especially among women from the Southern region, those who had twin or multiple births, and those who were older those who were from the poorer wealth quintile, and those who had children under 5 somewhat aligned with the prior studies. Our findings somewhat aligned with the findings mentioned in the National Strategic plan (2015–2019) of Jordan, in which the Southern region had higher child mortality rate than the national average (117). The regional variation could be due to many different reasons including quality of health (98) or other sociodemographic factors, underscoring further studies.

In the sample consisting only IPV victims, our findings regarding showing discrepancies of aORs between weighted and unweighted logistic regression models may have been many reasons. Although we found aORs of some variables in the unweighted models had higher odds, which may be overestimated and may have been biased (96, 97). However, we saw higher odds among some variables in the weighted model, underscoring further investigation. Although there was no known exact mechanism for these discrepancies, small sample size could be one of the reasons. The other reason could be inclusion of covariates. Despite our models being based on literature when selecting the covariates, there could be other covariates that could be missed, leading to discrepancies in aORs. All these findings require further investigations.

Our findings on marginal analyses of IPV survivors aligned with the findings from logistic regression. Our study found that women IPV survivors living in the rural areas is associated with increased likelihood of APOs compared to the urban residents (90). This finding was not surprising as those in the rural areas may have different challenges including limited access to health information, services, and ability to decide for their health care use (90). Those who live in the Southern region had 46-percentage point decrease in the likelihood of APOs compared to those living in the Central region. Regarding LBW, the IPV victims with primary education or who had secondary education, or those who had 3 or more children under age 5 years, those living in the rural, and those with other people made the decision had an increased likelihood of LBW. Contrarily, those who were from middle, richer, and the rural residents, and those who had ANC ≥8 visits had a decreased likelihood of delivering LBW babies. Regarding pregnancy loss, the IPV victims from the poorer or richer wealth quintiles, and those who had three or more children under age 5 years were less likely to experience pregnancy loss compared to their reference categories. Those living in the Southern region had a decreased likelihood of pregnancy loss compared to those in the Central region. Contrarily, those who had twins or more had higher likelihood of pregnancy loss compared to their refence category.

## Limitations

The strengths of this study were use of nationally representative data with a large sample size, transparent reporting of null findings, identification of important protective factors (wealth, SBA use), and risk factors (Northern region, multiple births) with clear theoretical and practical implications. However, there were several limitations to this study. We examined married women's data that had the most recent birth history, which limited our sample to 4,419. Although this sample size was adequate for data analyses, the inclusion criteria omitted a large proportion of available data. The survey collected self-reported data, which could lead to social desirability bias and under-reporting, especially for the questions related to IPV (58, 118). This limitation was largely mitigated by the survey methodology encouraging participants to provide honest and accurate responses, while maintaining their privacy and confidentiality (22, 37). Moreover, due to the cross-sectional nature of these data, causal relationships cannot be inferred. Furthermore, the temporal mismatch of data reported in this cross-sectional dataset could be a contributing factor for null results. More specifically, we examined lifetime IPV in the current study, which could have occurred before, during, or after the participant's most recent pregnancy. Additionally, the reported IPV prevalence in this sample was only 6.7%, which may have limited the power of the analysis to detect significant associations. Moreover, for the APOs, we combined all the APOs (including the pregnancy loss and livebirths) into a single variable, although each has different etiologies and risk factors. This may have led to potentially null association between APOs and IPV. To overcome this, we performed analyses among participants who experienced IPV for any APOs, pregnancy loss, and LBW (the other APOs did not have ample sample sizes for separate analyses). The subset analyses should be interpreted with caution due to small sample sizes and multicollinearity among subcategories for some variables. Additionally, some covariates (e.g., pregnancy loss among IPV victims with twins) showed large effect sizes with very wide confidence intervals, reflecting the model's overfitting due to small sample sizes. Therefore, extreme point estimates with wide CIs should be interpreted cautiously because they may not represent true population effects. Finally, in the current study, the DHS program asked respondents to report PTB data in months instead of days or weeks. Thus, the cut-off for PTB used in analyses was before 9 months instead of precise days (i.e., 259 days) or weeks (i.e., 37 weeks). This measurement imprecision may have biased estimates in the regression modeling the composite APO variable. Despite the mentioned limitations, this study provides critical information about the current situation regarding APOs and IPV in Jordan, a region with a traditionally patriarchal society with almost 9.5% miscarriages or 0.8% induced abortions, 0.2% stillbirths, 26.5% with LBW delivery, and 6.7% experiencing any IPV.

## Theoretical and practical implications

Presence of at least one-time IPV victimization had a potential for repeated cycle of violence (62), leading to stressful and traumatic conditions. Despite there being differences based on the individual's stages of stress and coping mechanism as posited by Selye's Theory of Stress (60), understanding individuals risk factors in this population is important. Additionally, interventions addressing early prevention of violence are urgently needed to stop or break the cycle of violence. Prevention of pregnancy complications, especially among IPV victims, is important and needs to be addressed urgently as PTB and LBW were found to be associated with decreased likelihood of education attainment and lower employment rate in adulthood (119).

Additionally, our finding regarding the significant association between wealthier women and lesser odds of APOs highlights an urgent need to implement affordable essential perinatal care for women from the poorer wealth quintiles. This finding may suggest the inequality in health care for women from the poorer wealth quintiles. An example of a potential solution may be adopting family health team approach for the perinatal women (120). Finally, the women in the Northern region had higher odds of APOs, underscoring a necessity to address health care access across the country. As of the 2015 census, only about 56% of Jordan's population has health insurance, which restricts access to health services for a substantial portion of the population (121). Health care coverage is concentrated in the Central and Eastern regions, which creates inequalities in health coverage within the Northern region and areas nationwide that are outside of these concentrated areas (122). This situation highlights the urgency of introducing interventions to prevent morbidity and mortality among mothers and infants, particularly those living in the Northern Region.

## Conclusion

IPV impacts the whole society beyond the individual woman. Recognizing the long-term impact of IPV on reproductive health is essential for providing comprehensive care to survivors, addressing both immediate and long-term health concerns. A deeper understanding about the relationship between IPV and APOs can also lead to developing tailored interventions and healthcare policies to support and empower those impacted by IPV.

## Data Availability

Publicly available datasets were analyzed in this study. This data can be found here: https://dhsprogram.com/data/available-datasets.cfm.
