# Supplementary material for: Association of adverse pregnancy outcomes and intimate partner violence survivorship: a cross-sectional survey
Source: Front Glob Womens Health. 2026 Feb 10;7:1616403. doi: 10.3389/fgwh.2026.1616403 (PMC12929379; doi:10.3389/fgwh.2026.1616403)
Supplement: Supplementary file 1 [file Table1.pdf]

Q26: Supplementary Table 1

Supplementary Table 1. Results from a series of survey weighted binary logistic regression models of adverse pregnancy outcomes and variance inflation factor (VIF) (All sampled women)

Variabls	APOs (n=3,932)		LBW (n=3,919)		Pregnancy loss (n=4,308)		VIF (APOs, pregnancy loss)	VIF (LBW)
	Unweighted aOR and 95% CI	Weighted aOR and 95%CI	Unweighted aOR and 95% CI	Weighted aOR and 95%CI	Unweighted aOR and 95% CI	Weighted aOR and 95%CI		
**Age in 5 years groups**								
15-19	Ref.							
20-24	0.90 (0.58, 1.39)	0.86 (0.41, 1.83)	0.70 (0.44, 1.12)	0.61 (0.29, 1.27)	2.11 (0.61, 7.30)	7.64 (1.29, 45.22) *	6.03	6.01
25-29	0.81 (0.53, 1.24)	0.63 (0.31, 1.29)	0.69 (0.44, 1.09)	0.55 (0.27, 1.13)	2.04 (0.60, 6.93)	5.73 (1.03, 31.92) *	8.97	8.95
30-34	0.80 (0.52, 1.23)	0.79 (0.37, 1.69)	0.79 (0.50, 1.25)	0.74 (0.37, 1.48)	4.23 (1.26, 14.25) *	11.98 (2.16, 66.58) **	8.83	8.82
35-39	0.87 (0.56, 1.34)	0.73 (0.34, 1.56)	0.81 (0.51, 1.29)	0.78 (0.37, 1.65)	5.13 (1.53, 17.22) **	17.3 (3.18, 94.10) **	7.39	7.36
40-44	0.91 (0.57, 1.45)	0.91 (0.41, 2.04)	0.79 (0.48, 1.31)	0.79 (0.38, 1.64)	7.68 (2.25, 26.13) **	25.75 (4.62, 143.66) ***	3.88	3.86
45-49	1.79 (0.79, 4.02)	1.22 (0.34, 4.43)	1.51 (0.66, 3.43)	0.76 (0.22, 2.63)	16.01 (4.23, 60.56) ***	66.47 (10.32, 428.38) ***	1.35	1.35
**Education**								
No education	Ref.							
Primary	1.57 (0.97, 2.52)	1.58 (0.66, 3.77)	1.26 (0.76, 2.10)	1.63 (0.65, 4.08)	1.97 (0.75, 5.20)	1.1 (0.29, 4.20)	4.06	4.05
Secondary	1.36 (0.88, 2.09)	1.15 (0.53, 2.48)	0.97 (0.61, 1.55)	1.12 (0.46, 2.74)	1.87 (0.75, 4.65)	1.06 (0.30, 3.70)	11.18	11.15
Higher	1.15 (0.73, 1.81)	0.95 (0.43, 2.07)	0.78 (0.48, 1.28)	0.91 (0.37, 2.27)	1.11 (0.43, 2.86)	0.59 (0.16, 2.11)	10.96	10.94
**Wealth quintiles**								
Poorest	Ref.	Ref.						
Poorer	0.74 (0.62, 0.89) **	0.87 (0.66, 1.15)	0.84 (0.68, 1.03)	0.96 (0.71, 1.30)	1.11 (0.81, 1.53)	0.87 (0.56, 1.37)	1.42	1.42
Middle	0.72 (0.59, 0.87) **	0.70 (0.53, 0.94) *	0.82 (0.65, 1.02)	0.75 (0.53, 1.05)	1.00 (0.71, 1.42)	1.04 (0.63, 1.72)	1.51	1.51
Richer	0.69 (0.55, 0.87) **	0.71 (0.49, 1.01)	0.69 (0.53, 0.90) **	0.58 (0.38, 0.88) *	1.03 (0.70, 1.52)	1.27 (0.72, 2.24)	1.53	1.53
Richest	0.61 (0.43, 0.85) **	0.50 (0.30, 0.83) **	0.57 (0.37, 0.86) **	0.48 (0.25, 0.90) *	1.17 (0.70, 1.96)	0.69 (0.31, 1.49)	1.31	1.31
**Place of residence**								
Urban	Ref.							
Rural	1.17 (0.99, 1.39)	1.20 (0.87, 1.67)	1.22 (1.01, 1.47) *	1.10 (0.80, 1.52)	1.03 (0.75, 1.40)	1.11 (0.65, 1.89)	1.08	1.08
**Region**								
Central	Ref.							
North	1.54 (1.32, 1.78) ***	1.44 (1.15, 1.80) **	1.33 (1.12, 1.58) *	1.38 (1.08, 1.75) **	0.96 (0.74, 1.25)	0.82 (0.56, 1.20)	1.31	1.31
South	1.32 (1.10, 1.59) **	1.20 (0.91, 1.58)	1.31 (1.06, 1.61) *	1.09 (0.82, 1.46)	0.93 (0.67, 1.30)	0.80 (0.53, 1.20)	1.37	1.37
**Currently employed**								
No	Ref.							
Yes	1.08 (0.85, 1.38)	1.26 (0.84, 1.89)	1.03 (0.78, 1.37)	1.22 (0.79, 1.88)	1.34 (0.91, 1.97)	1.52 (0.86, 2.69)	1.12	1.12
**Number of children under age 5 years**								
No child	Ref.							
One or two children	0.94 (0.69, 1.29)	0.92 (0.58, 1.48)	0.91 (0.63, 1.31)	0.97 (0.56, 1.66)	0.11 (0.08, 0.14) ***	0.10 (0.07, 0.16) ***	4.00	4.09
Three or more children	0.82 (0.58, 1.16)	0.70 (0.41, 1.20)	0.89 (0.60, 1.32)	0.92 (0.50, 1.70)	0.04 (0.03, 0.08) ***	0.05 (0.02, 0.10) ***	4.04	4.13
**Child born is twins or multiple**								
No, single birth	Ref.							
Yes	4.52 (2.62, 7.81) ***	3.68 (1.70, 7.96) **	7.87 (4.68, 13.23) ***	5.48 (2.54, 11.81) ***	1.95 (0.97, 3.93)	2.36 (0.88, 6.33)	1.03	1.03
**Number of ANC visit**								
<8 visits	Ref.							

≥8 visits	0.90 (0.78, 1.02)	0.86 (0.71, 1.05)	0.89 (0.77, 1.04)	0.85 (0.69, 1.05)	NA	NA	1.06	1.06
**Use of skilled birth attendants**								
No	Ref.							
Yes	0.19 (0.02, 1.75)	0.06 (0.01, 0.64) *	0.08 (0.01, 0.74)	0.03 (0.00, 0.27) **	NA	NA	1.01	1.01
**Presence of STI in the past 12 months**								
No	Ref.							
Yes	0.99 (0.78, 1.24)	0.90 (0.63, 1.30)	1.11 (0.85, 1.44)	0.87 (0.58, 1.32)	1.03 (0.69, 1.53)	1.44 (0.77, 2.70)	1.02	1.02
**Smoking cigarettes or tobacco**								
No	Ref.							
Yes	1.1 (0.87, 1.39)	1.32 (0.91, 1.91)	1.05 (0.80, 1.37)	0.97 (0.65, 1.46)	1.14 (0.79, 1.65)	1.63 (1.02, 2.58) *	1.04	1.03
**Big problem for medical help or distance to health facility**								
No	Ref.							
Yes	0.88 (0.74, 1.05)	0.91 (0.68, 1.22)	0.97 (0.80, 1.18)	1.04 (0.73, 1.47)	0.94 (0.69, 1.29)	1.31 (0.80, 2.15)	1.06	1.06
**Decision making**								
Respondent alone	Ref.							
Respondent and her partner	0.97 (0.83, 1.14)	1.06 (0.81, 1.39)	0.91 (0.76, 1.10)	0.99 (0.73, 1.33)	1.03 (0.78, 1.36)	0.78 (0.51, 1.19)	1.29	1.29
Her partner alone	1.19 (0.90, 1.56)	1.33 (0.86, 2.08)	1.04 (0.77, 1.41)	1.20 (0.73, 1.97)	0.95 (0.57, 1.57)	1.07 (0.56, 2.06)	1.27	1.27
Other people	0.79 (0.14, 4.41)	1.77 (0.21, 15.11)	0.7 (0.08, 6.11)	2.3 (0.22, 24.58)	1.25 (0.13, 12.37)	0.34 (0.02, 5.44)	1.02	1.02
**Any IPV**								
No	Ref.							
Yes	1.19 (0.91, 1.55)	1.04 (0.69, 1.57)	1.1 (0.82, 1.49)	1 (0.64, 1.54)	1.6 (1.08, 2.37) **	1.03 (0.59, 1.82)	1.02	1.02
Constant	3.97 (0.39, 40.16)	14.52 (1.02, 205.83)	6.63 (0.64, 69.03)	18.91 (1.27, 281.98) *	0.1 (0.02, 0.48)	0.07 (0.01, 0.52) *		
Mean VIF							3.18	3.18

Note. APOs= Adverse Pregnancy Outcomes; LBW= Low Birth Weight; aOR= adjusted Odds Ratio; 95% CI= 95% Confidence Interval; VIF= Variance Inflation Factor; *=p<0.05, **=p <0.01, ***=p<0.001
