# Supplementary material for: Association of adverse pregnancy outcomes and intimate partner violence survivorship: a cross-sectional survey
Source: Front Glob Womens Health. 2026 Feb 10;7:1616403. doi: 10.3389/fgwh.2026.1616403 (PMC12929379; doi:10.3389/fgwh.2026.1616403)
Supplement: Supplementary file 2 [file Table2.pdf]

Q26: Supplementary Table 2

Supplementary Table 2: Results from a series of survey weighted binary logistic regression models of adverse pregnancy outcomes (APOs), low birth weight (LBW), and pregnancy loss among women IPV survivors

Variables	APOs (n=239)		LBW (n=236)		Pregnancy loss (n=279)		VIF (APOs, pregnancy loss)	VIF (LBW)
	Unweighted aOR (95% CI)	Weighted aOR (95% CI)	Unweighted aOR (95% CI)	Weighted aOR (95% CI)	Unweighted aORs (95% CI)	Weighted aOR (95% CI)		
**Age in 5 years groups**								
15-19	Ref.				Empty	Empty		
20-24	0.75 (0.04, 15.94)	0.34 (0.01, 17.44)	0.08 (0.00, 2.37)	0.17 (0, 140.93)	0.06 (0.01, 0.53) *	0.00 (0.00, 0.09) **	15.34	15.33
25-29	0.84 (0.04, 17.10)	0.42 (0.01, 21.19)	0.10 (0.00, 2.98)	0.37 (0, 270.72)	0.05 (0.01, 0.45) **	0.00 (0.00, 0.04) ***	27.16	27.12
30-34	0.93 (0.04, 19.75)	0.56 (0.01, 33.20)	0.12 (0.00, 3.38)	0.35 (0, 269.01)	0.04 (0, 0.31) **	0.00 (0.00, 0.06) ***	29.58	29.52
35-39	1.87 (0.09, 39.36)	0.87 (0.02, 41.83)	0.33 (0.01, 9.15)	0.85 (0, 686.8)	0.10 (0.01, 0.83) *	0.00 (0.00, 0.10) **	22.67	22.32
40-44	0.55 (0.02, 12.56)	0.07 (0.00, 3.69)	0.09 (0.00, 2.99)	0.06 (0, 62.96)	0.09 (0.01, 0.92) *	0.01 (0.00, 0.19) **	11.62	11.61
45-49	2.08 (0.03, 149.67)	0.66 (0.00, 100.25)	-	-	,-	-	2.22	2.22
**Education**								
No education	Ref.							
Primary	1.23 (0.14, 10.90)	1.29 (0.18, 8.98)	3.71 (0.41, 33.40)	50.70 (2.55, 1008.76) *	1.05 (0.09, 12.48)	1.43 (0.04, 50.32)	5.50	5.49
Secondary	1.14 (0.15, 8.91)	1.31 (0.23, 7.57)	3.39 (0.42, 27.61)	23.92 (1.05, 544.86) *	2.12 (0.25, 18.05)	1.66 (0.07, 41.11)	12.63	12.62
Higher	0.70 (0.08, 5.90)	0.38 (0.05, 2.65)	1.05 (0.11, 9.62)	5.30 (0.20, 140.08)	1.04 (0.09, 12.04)	3.22 (0.10, 102.35)	11.37	11.36
**Wealth quintile**								
Poorest	Ref.							
Poorer	1.10 (0.53, 2.29)	0.90 (0.32, 2.54)	1.50 (0.66, 3.42)	1.44 (0.51, 4.07)	0.17 (0.04, 0.76) *	0.03 (0.00, 0.23) **	1.51	1.52
Middle	0.71 (0.30, 1.68)	0.25 (0.06, 1.05)	0.46 (0.16, 1.31)	0.20 (0.05, 0.78) *	0.57 (0.19, 1.69)	1.30 (0.39, 4.36)	1.53	1.54
Richer	0.42 (0.14, 1.29)	0.32 (0.04, 2.42)	0.64 (0.16, 2.45)	0.15 (0.03, 0.72) *	0.94 (0.22, 4.01)	0.14 (0.02, 1.30)	1.50	1.51
Richest	0.34 (0.03, 3.44)	0.47 (0.05, 4.58)	1.53 (0.14, 17.02)	1.85 (0.20, 17.15)	2.04 (0.22, 18.53)	1.25 (0.18, 8.78)	1.25	1.25
**Place of residence**								
Urban	Ref.							
Rural	2.57 (1.12, 5.91) *	7.72 (2.42, 24.60) **	3.69 (1.56, 8.71) **	15.04 (3.42, 66.16) ***	1.27 (0.45, 3.6)	0.98 (0.25, 3.79)	1.22	1.23
**Region**								
Central	Ref.							
North	2.11 (1.10, 4.02) *	1.98 (0.76, 5.15)	1.94 (0.91, 4.11)	1.95 (0.52, 7.38)	2.10 (0.78, 5.7)	3.21 (0.88, 11.76)	1.32	1.32
South	0.43 (0.15, 1.18)	0.14 (0.02, 0.82) *	0.97 (0.32, 2.92)	0.17 (0.01, 1.95)	4.88 (1.39, 17.07) *	6.09 (1.10, 33.73) *	1.40	1.40
**Currently employed**								
No	Ref.							
Yes	1.88 (0.56, 6.33)	3.33 (0.71, 15.53)	1.32 (0.32, 5.48)	6.84 (0.96, 48.97)	0.24 (0.02, 2.28)	0.33 (0.01, 8.27)	1.26	1.26
**Number of children under age 5 years**								
No child	Ref.							
One or two children	1.07 (0.32, 3.59)	2.32 (0.42, 12.90)	0.94 (0.21, 4.27)	4.43 (0.75, 26.15)	0.14 (0.05, 0.41) ***	0.04 (0.01, 0.23) ***	3.68	3.67
Three or more children	1.11 (0.29, 4.29)	3.56 (0.53, 23.80)	1.68 (0.33, 8.52)	13.66 (2.26, 82.52) **	0.05 (0.01, 0.34) **	0.01 (0.00, 0.14) **	3.55	3.55
**Child born is twins or multiple**	-	-	-	-	-	-	-	-

No, single birth	Ref.							
Yes	-	-	-	-	7.03 (0.84, 58.66)	66.15 (6.19, 706.50) **	1.11	1.11
**Number of ANC visit**								
<8 visits	Ref.	Ref.						
≥8 visits	0.61 (0.34, 1.10)	0.42 (0.17, 1.01)	0.54 (0.28, 1.06)	0.21 (0.08, 0.51) ***	-	-	1.08	1.08
**Use of skilled birth attendants**	-	-	-		-	-		
No	Ref.							
Yes	-	-	-	-	-	-	-	-
**Presence of STI in the past 12 months**								
No	Ref.							
Yes	0.58 (0.23, 1.51)	1.82 (0.50, 6.63)	0.49 (0.15, 1.61)	2.88 (0.8, 10.41)	0.71 (0.18, 2.79)	0.24 (0.04, 1.59)	1.12	1.12
**Smoking cigarettes or tobacco**								
No	Ref.							
Yes	0.65 (0.24, 1.75)	0.36 (0.09, 1.38)	0.99 (0.29, 3.37)	0.43 (0.07, 2.52)	2.00 (0.58, 6.91)	2.53 (0.63, 10.11)	1.17	1.17
**Big problem for medical help or distance to health facility**								
No	Ref.							
Yes	1.33 (0.68, 2.60)	0.76 (0.30, 1.90)	0.88 (0.42, 1.87)	0.59 (0.16, 2.11)	0.59 (0.22, 1.62)	1.72 (0.36, 8.18)	1.2	1.2
**Decision making**								
Respondent alone	Ref.							
Respondent and her partner	1.59 (0.82, 3.09)	1.71 (0.65, 4.50)	1.73 (0.77, 3.90)	2.51 (0.96, 6.56)	1.46 (0.51, 4.15)	2.3 (0.68, 7.78)	1.43	1.43
Her partner alone	2.66 (1.01, 7.03) *	2.68 (0.75, 9.64)	3.08 (1.03, 9.21)	7.21 (1.7, 30.59) **	1.82 (0.45, 7.31)	2.05 (0.29, 14.46)	1.33	1.33
Other people	-	-	-	-	-	-	-	-
**Constant**	0.57 (0.02, 21.75)	0.79 (0.01, 45.64)	0.54 (0.02, 15.25)	0.01 (0, 29.49)	4.71(0.32, 69.86)	141.45 (0.62, 32179.35)	-	-
**Mean VIF**							6.34	6.32

Note. APOs= Adverse Pregnancy Outcomes; LBW= Low Birth Weight; aOR= adjusted Odds Ratio; 95% CI= 95% Confidence Interval; VIF= Variance Inflation Factor; *=p<0.05, **=p <0.01, ***=p<0.001
